# Health profile for Danish adults with activity limitation: a cross-sectional study

**DOI:** 10.1186/s12889-017-4532-0

**Published:** 2017-07-24

**Authors:** Nina Føns Johnsen, Michael Davidsen, Susan Ishøy Michelsen, Knud Juel

**Affiliations:** National Institute of Public Health, University of Southern Denmark, Øster Farimagsgade 5A, DK-1353 Copenhagen K, Denmark

**Keywords:** Disability, Activity limitation, Health, Health behaviour, Social relations

## Abstract

**Background:**

Studies have indicated that people with disabilities die earlier and may experience a poorer health than the general population. This study investigated 31 factors related to health and well-being, health behaviour and social relations among Danish adults with activity limitation (AL).

**Methods:**

This study was based on data from the Danish Health and Morbidity Survey (DHMS) 2013 where 25,000 men and women aged 16 years or older were selected randomly from the adult Danish population. A total of 14,265 individuals answered the self-administered questionnaire including 100 questions on health-related quality of life, health behaviour, morbidity, consequences of illness and social relations. Based on an international standard question on AL, 888 individuals (6%) were defined as having profound AL and 4180 (29%) as having some AL. Multiple logistic regression analyses were used to analyse the associations between activity limitation and 31 indicators of health. The results were presented as relative risks 95% confidence intervals.

**Results:**

Twenty-eight of 31 indicators showed consistently poorer health and well-being, health behaviour and social relations among individuals with AL as compared to individuals without AL. The increased relative risks were in a range of 7-661% the risk among individuals without AL. An example is obesity where RR (95% CI) was 2.07 (1.82–2.37).

Only contact with internet friends was significantly higher among individuals with AL as compared to individuals with no AL. There was no association between alcohol and AL and no association between fast food and some AL.

**Conclusion:**

Danish adults with AL experience a poorer health and well-being, and have an unhealthier lifestyle and poorer social relations than adults without AL. People with activity limitation should be prioritized in public health and efforts done to secure availability and flexibility of health care services and primary prevention programs. Policies should address accessibility, availability and affordability of health care and health behaviour among people with activity limitation.

**Electronic supplementary material:**

The online version of this article (doi:10.1186/s12889-017-4532-0) contains supplementary material, which is available to authorized users.

## Background

Health can be described as a state of complete physical, mental, and social well-being and not merely the absence of disease or infirmity [1], and health is fundamental for all people’s well-being and essential for daily activity and (social) participation. The United Nations Convention on the Rights of Persons with Disabilities reinforces the rights of persons with disabilities to attain the highest standard of health care, without discrimination [1] and the WHO states that ‘*Equity in health implies that ideally everyone should have a fair opportunity to attain their full health potential, and more pragmatically, that none should be disadvantaged from achieving this potential, if it can be avoided.’* [2]. According to the WHO and the International Classification of Functioning (ICF), ‘disability’ is an umbrella term, covering bodily impairments, activity limitations, and participation restrictions [3].

Unfortunately, the morbidity and mortality of people with disabilities are considerably higher than in the general population [4–12]. A study from the UK indicated that people with intellectual disabilities die 13–20 years earlier than the general population [7], and although the literature is sparse and both mortality and health probably differ by type and extent of disability, this study highlights the need for further investigations. The early death of people with disabilities may result from a primary health condition, but some of the inequalities in longevity may also be caused by secondary health conditions, suboptimal use of health care and unhealthy lifestyle. Studies have shown that secondary health problems are very common among people with intellectual disability [13, 14].

In 2011, the World Report on Disability emphasized that there are systematic barriers to the health and longevity of people with disabilities, and more importantly, that many of these barriers are modifiable [1]. Examples of barriers to overcome are a physical inaccessible environment and infrastructure, unmet needs for assistance (persons or tools) and health care, higher economic expenses (medicine, transport, food etc.), and discrimination in the labour market.

Very few international, peer-reviewed studies on the over-all health of people with disabilities have been published; to our knowledge, no European studies have yet been published on this topic. However, three health profile studies from the US and Korea (where the study by Havercamp et al., 2015 represents five other studies on the US Behavior Risk Factor Surveillance study [13, 15–19]) show that people with disabilities have a range of unhealthy behaviours like physical inactivity, smoking, obesity; they have inadequate emotional support, a range of chronic health conditions and inadequate medical care [16, 20, 21]. These studies have been supported by single-indicator studies showing that people with disability more often are physically inactive [22–24], obese [25, 26], smokers [27, 28] and experience life dissatisfaction and inadequate social support [29] as compared to people without disability.

In addressing the Danish National Government’s focus on equality in health, the aim of this study was to investigate 31 factors related to health and well-being, health behaviour and social relations among Danes with activity limitation (AL) and to compare the occurrence of these factors to the occurrence among individuals with no AL.

## Methods

### Basis population

We used data from the Danish Health and Morbidity Survey 2013. This survey is based on a nationally representative sample of 25,000 individuals aged 16 years or older who were resident in Denmark on January 1st, 2013. The sample was drawn randomly from the Central Person Registry using the unique personal registration number. In February 2013 the potential participants received a letter of introduction that briefly described the purpose and content of the survey and that they could either fill in an enclosed printed questionnaire or a web-questionnaire. It was emphasized that participation was voluntary. Data were collected from February to April 2013, and in this period a maximum of two postal reminders were sent. The questionnaire was readable by Jaws (the leading screen reader) and was therefore accessible for visually impaired people and people with dyslexia. The questionnaire was also accessible for people with different types of cognitive limitations (ADHD, Asperger’s syndrome etc.), however, people with more severe cognitive limitations may have needed assistance from a relative or personal assistant to recall and report (i.e. to answer the questionnaire). The questionnaire included 100 questions regarding health and well-being, morbidity, physical functioning, health behaviour, medical use, social relations in addition to working environment and housing conditions. The Danish Health and Morbidity Surveys have previously been described in detail [30, 31]. A total of 14,265 individuals filled in the questionnaire, corresponding to a participation proportion of 57%.

### Identification of individuals with AL

Individuals with AL were defined on the basis of a standardized and internationally validated question for measuring AL [32]: “Have you within the latest 6 months, due to health problems or illness, been limited in carrying out activities that people usually do - have you experienced profound limitation, some limitation, or no limitation?” The answers to this question were used directly in a variable describing the extent of AL among the study participants: Profound AL, Some AL, and No AL.

### Health indicators

In this section the justification and the definition of the 31 health indicators are described. A more detailed description of the operationalization of variables is shown in the Additional file 1: Table S1.

#### Health and well-being

Regarding health and well-being, 16 indicators were selected: Poor self-perceived health was used as an indicator of overall health, and poor dental status was used as an indicator of poor teeth and mouth health and hygiene. The symptom indicators were: pain or discomfort in the musculoskeletal system, headache, fatigue, dyssomnia, psychological symptoms and stress (Cohens perceived stress scale). Medicine was included as six indicators of over-the-counter or prescription-only of: hypnotics/sedatives, analgesics, and laxatives. Last, two indicators of sexual health were included: no sexual contact (within the past year) and unsatisfied with their sexual life. Data on self-perceived health and stress were based on WHO’s recommended question on self-perceived health [33] and Cohen’s Perceived Stress Scale [34].

#### Health behaviour

Regarding health behaviour, a total of eight indicators were selected: No daily intake of vegetables and weekly intake of fast food were used as simple measures of an unhealthy diet. Risk factors for morbidity and mortality were: daily smoking and heavy smoking (at least 15 cigarettes per day), alcohol intake above the low-risk and high-risk limits (7/14 units per week for women and men, respectively; 14/21 units per week for women and men, respectively), sedentary leisure time activity, and overweight (BMI ≥ 25) and obesity (BMI ≥ 30). Data on physical activity were based on the Saltin-Grimsby Physical Activity Level Scale [35].

#### Social relations

Regarding social relations, a total of six indicators were selected: infrequent contact with family, infrequent contact with friends, infrequent contact with internet friends, feeling alone, no one to talk to, and no practical help.

### Statistical methods

Calibration weights developed by Statistics Denmark were used in order to accommodate for non-response. The prevalence of each health indicator was computed in each AL group. Multiple logistic regression analyses were used to analyse the associations between AL and the 31 health indicators controlling for age and gender. The results are presented as relative risks (RR) with 95% confidence intervals (CI). SAS version 9.3 (SAS Institute, North Carolina) procedure GENMOD was used for the logistic regression analyses.

## Results

### Characteristics of study population

A total of 888 individuals (6%) were defined as having profound AL and 4180 (29%) as having some AL. The characteristics of the study population are shown in Table 1.

**Table 1:** Characteristics of individuals in the Danish Health and Morbidity survey, 2013

	Activity limitation							
	No		Some		Profound		Total	
	Percentage^a^	No.	Percentage^a^	No.	Percentage^a^	No.	Percentage^a^	No.
Sex								
Men	51.6	4072	45.3	1758	49.5	417	49.6	6247
Women	48.4	4585	54.7	2422	50.5	471	50.4	7478
Age (years)								
16–44	51.3	3613	36.7	1202	30.3	212	45.4	5027
45–64	31.7	3184	35.4	1618	38.5	347	33.3	5149
65–74	11.7	1373	14.7	784	14.2	170	12.8	2327
≥ 75	5.2	487	13.2	576	16.9	159	8.4	1222
Education								
Attending educational institution	12.9	894	8.2	267	4.5	31	10.9	1192
Basic school	6.2	548	11.5	459	17.6	137	8.5	1144
Upper secondary or vocational education	35.1	3019	39.1	1563	41.0	339	36.7	4921
Short further education	7.5	633	7.7	301	6.2	51	7.5	985
Bachelor degree	20.7	1838	18.2	772	16.9	141	19.7	2751
Master degree	13.7	1072	9.0	342	5.8	48	11.8	1462
Other	3.9	311	6.3	218	8.0	56	4.9	585
Occupation								
Employed	57.4	4868	40.9	1713	23.7	225	50.0	6806
Unemployed	4.5	320	4.3	150	5.7	41	4.6	511
Student	15.9	1109	9.5	317	5.8	41	13.3	1467
Disability pensioner	0.8	48	8.3	267	17.5	130	4.2	445
Early retirement pensioner	3.0	311	3.5	185	1.3	15	3.0	511
Pensioner	17.0	1860	28.0	1360	31.2	329	21.4	3549
Others out of job	1.4	89	5.4	167	14.8	104	3.5	360
Cohabitation								
Cohabitant	62.0	5874	57.6	2716	52.2	532	60.0	9122
Single	38.0	2.783	42.4	1464	47.8	356	40.0	4603
Diseases								
Asthma	5.0	416	9.2	370	12.7	103	6.8	889
Allergy	19.6	1609	20.8	844	20.7	166	20.0	2619
Diabetes	3.0	296	7.5	302	11.0	100	4.9	698
Hypertension	12.6	1300	22.8	1016	27.5	252	16.7	2568
Myocardial infarct	0.2	21	1.6	70	3.7	32	0.8	132
Angina pectoris	0.3	30	2.5	102	4.3	34	1.2	166
Apoplexia	0.4	38	2.4	100	6.1	57	1.4	195
Chronic bronchitis, emphysema	1.3	121	7.1	299	12.1	110	3.8	530
Arthrosis	9.7	980	29.2	1319	37.5	333	17.5	2632
Rheumatoid arthritis	1.8	170	8.7	359	12.9	105	4.6	634
Osteoporosis	1.1	126	5.1	229	9.6	81	2.9	436
Cancer	1.0	102	4.2	203	5.9	65	2.3	370
Migraine, frequent headache	9.6	824	19.7	756	23.7	189	13.6	1769
Herniated disc	5.1	452	20.4	838	31.5	264	11.6	1554
Cataract	2.2	215	5.4	238	6.8	65	3.5	518
Tinnitus	9.6	872	15.4	657	15.9	142	11.8	1671
At least one disease	14.5	745	21.5	3292	52.5	4699	37.4	4722

^a^Weighted percentages

**Fig. 1:**
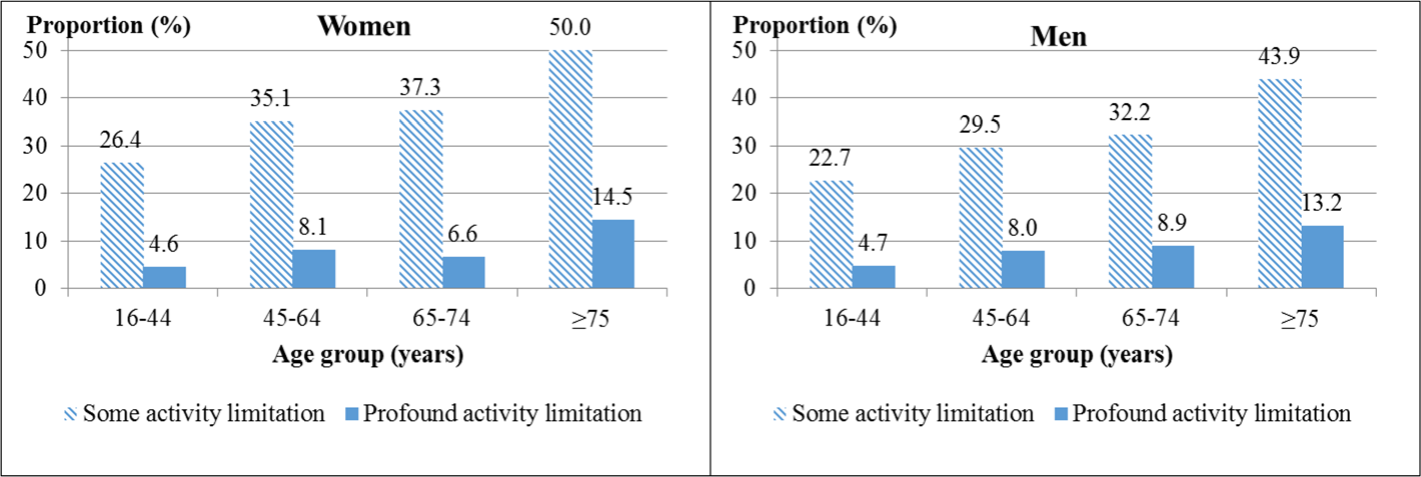
Age distribution in activity limitation groups

A total of 14,265 women and men participated in SUSY-2013 and 13,725 answered the question on AL. Of these, 7478 or 50.4% were women and 6247 or 49.6% were men, and 862 individuals reported profound AL, 4078 reported some AL and 8518 reported no AL. The median (25, 75 percentile) age was 51 (36, 65) among women and 53 (37, 66) among men. Figure 1 shows the age distribution in AL groups.

Among individuals with profound AL, 16.9% had a bachelor degree and 5.8% had a master degree. Among individuals with some AL, 18.2% had a bachelor degree and 9.0% had a master degree. Among individuals with no AL, the corresponding numbers were 20.7% and 13.7%, respectively.

Among individuals with profound AL, 23.7% were employed, among individuals with some AL 40.9% were employed and among individuals with no AL 57.4% were employed. In contrast, 17.5% of individuals with profound AL received disability pension, while 8.3% and 0.8% of individuals with some or no AL received disability pension.

Among individuals with profound AL 52.2% were cohabitating; among individuals with some or no AL 57.6% and 62.0% were cohabitating, 12.7% of individuals with profound AL reported asthma as compared to 9.2% of individuals with some AL and 5.0% of individuals with no AL. Similar proportions were found for diabetes, chronic bronchitis, and emphysema. Among individuals with profound AL 37.5% reported rheumatoid arthritis as compared to 29.2% and 9.7% among individuals with some and no AL, respectively. Among individuals with profound AL 31.5% reported herniated discs as compared to 20.4% and 5.1% among individuals with some and no AL, respectively. A total of 52.5% of individuals with profound AL reported having at least one disease as compared to 21.5% of individuals with some AL and 14.5% of individuals with no AL.

### Health profile

#### Health and well-being

**Fig. 2:**
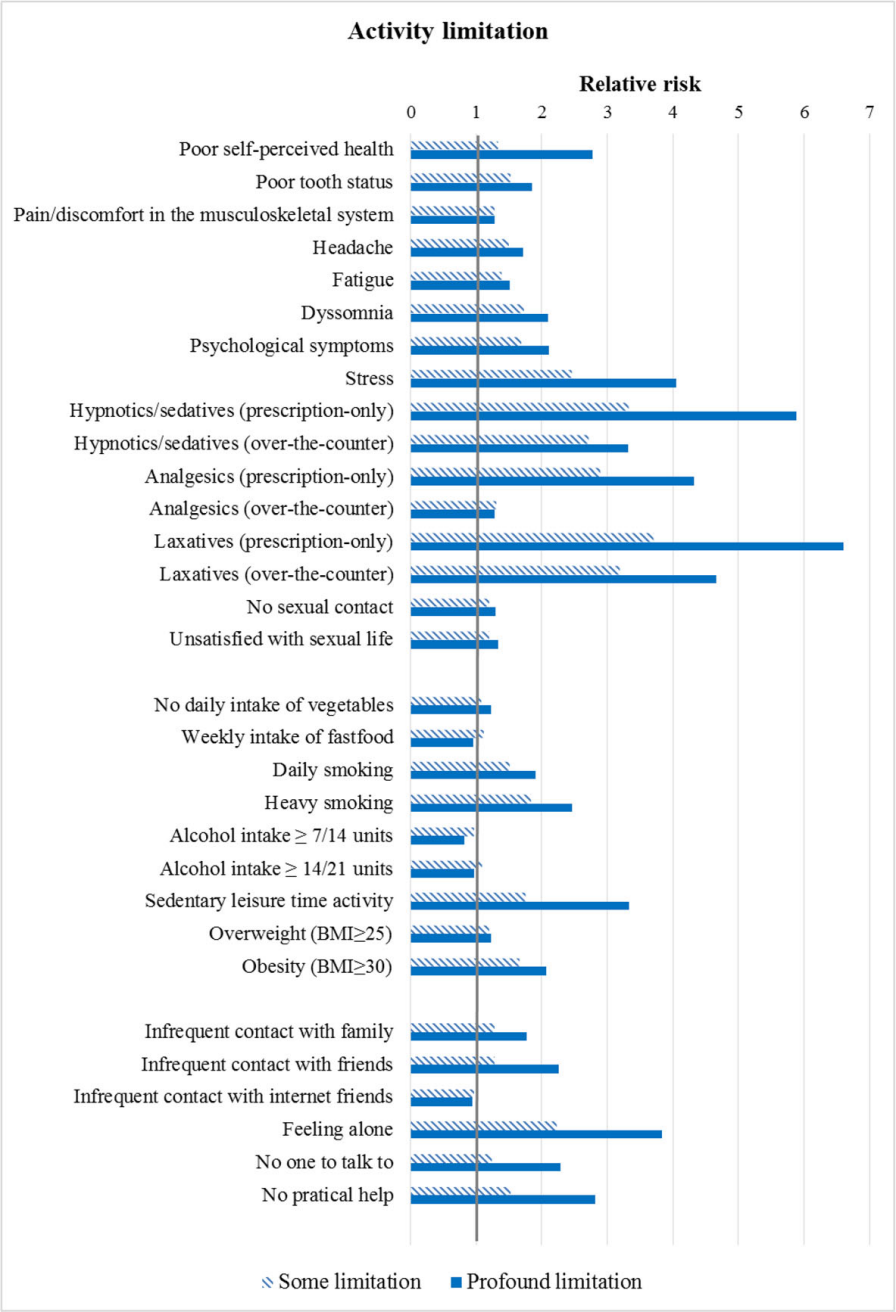
Relative risk of health indicators among individuals with some (striped) or profound (filled) activity limitation (reference = no activity limitation)

In Table 2 and Fig. 2 the proportions of individuals with a specific health indicator are shown for each level of AL. Furthermore, the RR of each indicator of health and well-being is shown for each level of AL.

**Table 2:** Percentages and relative risks (95% confidence intervals) for indicators of health and well-being

Activity limitation	No		Some		Profound	
	Percentage with health indicator^a^	RR (95% CI)^b^	Percentage with health indicator^a^	RR (95% CI)^b^	Percentage with health indicator^a^	RR (95% CI)^b^
Health and well-being						
Poor self-perceived health	2.2	1	27.4	1.33 (1.32–1.37)	64.8	2.78 (2.50–3.00)
Poor dental status	9.5	1	21.8	1.52 (1.40–1.65)	31.1	1.85 (1.68–2.03)
Pain or discomfort in musculoskeletal system	71.4	1	92.1	1.27 (1.25–1.29)	93.5	1.28 (1.26–1.31)
Headache	31.2	1	44.6	1.50 (1.43–1.57)	49.6	1.72 (1.61–1.83)
Fatigue	53.2	1	74.7	1.39 (1.35–1.43)	83.5	1.51 (1.47–1.56)
Dyssomnia	30.0	1	51.9	1.72 (1.64–1.80)	62.9	2.10 (1.98–2.23)
Psychological symptoms	29.9	1	49.5	1.69 (1.61–1.76)	61.6	2.11 (1.99–2.24)
Stress	14.0	1	33.7	2.46 (2.29–2.64)	55.3	4.05 (3.74–4.38)
Hypnotics/sedatives (prescription-only)	4.1	1	14.9	3.32 (2.92–3.77)	27.0	5.89 (5.08–6.84)
Hypnotics/sedatives (over-the-counter)	0.8	1	2.1	2.71 (1.97–3.71)	2.6	3.32 (2.10–5.26)
Analgesics (prescription-only)	11.9	1	35.7	2.89 (2.69–3.10)	54.0	4.32 (3.98–4.69)
Analgesics (over-the-counter)	34.7	1	43.0	1.30 (1.24–1.36)	40.6	1.28 (1.19–1.39)
Laxatives (prescription-only)	2.9	1	11.5	3.70 (3.19–4.30)	20.3	6.61 (5.53–7.89)
Laxatives (over-the-counter)	0.4	1	1.2	3.19 (2.05–4.97)	1.8	4.66 (2.59–8.40)
No sexual contact	26.2	1	37.8	1.19 (1.13–1.25)	51.4	1.30 (1.24–1.38)
Unsatisfied with sexual life	14.2	1	18.9	1.40 (1.29–1.53)	22.0	1.62 (1.41–1.85)

^a^Unadjusted; ^b^Adjusted for sex and age

All the indicators on health and well-being showed that individuals with AL had poorer health and well-being compared to individuals with no AL. A larger proportion of individuals with AL reported poor self-perceived health, poor dental status; pain or discomfort in musculoskeletal system; headache; fatigue; dyssomnia; psychological symptoms; stress; use of prescription-only and over-the-counter hypnotics/sedatives, analgesics and laxatives; no sexual contact and dissatisfaction with sexual life as compared to individuals with no AL.

The range in RR’s was 1.19–3.70 among individuals with some AL and 1.28–6.61 among individuals with profound AL. Most of the RR’s were in the order of 1.5–2, but use of medicine had markedly higher RR’s. Examples are poor dental status and dyssomnia with RR’s (95% CI) of 1.52 (1.40–1.65) and 1.72 (1.64–1.80) among individuals with some AL, and 1.85 (1.68–2.03) and 2.10 (1.98–2.23) for individuals with profound AL.

There seemed to be a trend towards poorer health and well-being with more AL, as individuals with profound AL had poorer health and well-being compared to individuals with some AL who again had poorer health and well-being than individuals with no AL.

#### Health behaviour

In Table 3 and Fig. 2 the proportions of individuals with a specific health indicator are shown for each level of AL. Furthermore, for each level of AL the RR of each indicator of health behaviour is shown.

**Table 3:** Percentages and relative risks (95% confidence intervals) for indicators of health behavior

Activity limitation	No		Some		Profound	
	Percentage with health indicator^a^	RR (95% CI)^b^	Percentage with health indicator^a^	RR (95% CI)^b^	Percentage with health indicator^a^	RR (95% CI)^b^
Health behaviour						
No daily intake of vegetables	39.4	1	43.2	1.07 (1.03–1.12)	52.7	1.23 (1.15–1.30)
Weekly intake of fast food	14.1	1	12.5	1.12 (1.03–1.23)	10.1	0.96 (0.80–1.16)
Daily smoking	14.4	1	21.4	1.51 (1.39–1.63)	27.0	1.91 (1.69–2.14)
Heavy smoking	6.4	1	11.1	1.83 (1.63–2.06)	15.0	2.46 (2.07–2.92)
Alcohol intake above 7/14 units/week	20.5	1	19.8	0.97 (0.89–1.04)	16.7	0.82 (0.70–0.95)
Alcohol intake above 14/21 units/week	8.1	1	8.5	1.08 (0.95–1.22)	7.8	0.97 (0.76–1.23)
Sedentary leisure time activity	11.1	1	20.8	1.75 (1.61–1.91)	41.7	3.34 (3.03–3.68)
Overweight	42.4	1	53.4	1.20 (1.15–1.24)	57.6	1.23 (1.16–1.31)
Obesity	10.7	1	18.2	1.65 (1.50–1.80)	23.3	2.07 (1.82–2.37)

^a^Unadjusted; ^b^Adjusted for sex and age

Except for alcohol and fast food, all the indicators on health behaviour showed that individuals with AL had poorer health behaviour compared to individuals with no AL. A larger proportion of individuals with AL reported having no daily intake of vegetables, weekly intake of fast food, daily smoking, heavy smoking, sedentary leisure time activity and being overweight or obese as compared to individuals with no AL. Only among individuals with some AL, a larger proportion had a weekly intake of fast food; among individuals with profound AL there was no association with weekly intake of fast food. Regarding alcohol intake, a smaller proportion of individuals with profound AL had an intake above the low-risk limits of 7 and 14 units per week for women and men, respectively.

The range in RR’s was 0.97–1.83 among individuals with some AL and 0.82–3.34 among individuals with profound AL (with alcohol and fast food having some RR’s below 1). Most of the RR’s were in the order of 1–2. Examples are daily smoking and overweight with RR’s (95% CI) of 1.51 (1.39–1.63) and 1.20 (1.15–1.24) for individuals with some AL, and 1.91 (1.69–2.14) and 1.23 (1.16–1.31) for individuals with profound AL.

Again, there seemed to be a trend towards poorer health behaviour with more AL.

#### Social relations

In Table 4 and Fig. 2 the proportions of individuals with a specific health indicator are shown for each level of AL. Furthermore, for each level of AL the RR of each indicator of social relations is shown.

**Table 4:** Percentages and relative risks (95% confidence intervals) for indicators of social relations

Activity limitation	No		Some		Profound	
	Percentage with health indicator^a^	RR (95% CI)^b^	Percentage with health indicator^a^	RR (95% CI)^b^	Percentage with health indicator^a^	RR (95% CI)^b^
Social relations						
Infrequent contact with family	6.6	1	8.2	1.27 (1.11–1.45)	11.7	1.77 (1.46–2.15)
Infrequent contact with friends	3.3	1	4.4	1.27 (1.06–1.53)	8.9	2.46 (1.93–3.12)
Infrequent contact with internet friends	76.2	1	75.0	0.97 (0.95–0.99)	73.2	0.95 (0.92–0.99)
Feeling alone	3.6	1	8.0	2.23 (1.91–2.60)	13.5	3.84 (3.15–4.68)
No one to talk to	3.2	1	4.1	1.24 (1.03–1.50)	8.0	2.29 (1.79–2.94)
No practical help	2.3	1	4.0	1.52 (1.24–1.87)	7.7	2.82 (2.17–3.68)

^a^Unadjusted; ^b^Adjusted for sex and age

Except for infrequent contact with internet friends, all the indicators on social relations showed that individuals with AL had less contact with their social relations compared to individuals with no AL. A larger proportion of individuals with AL reported having infrequent contact with family and friends, feeling alone no one to talk to and no practical help as compared to individuals with no AL. Among individuals with some and profound AL, a smaller proportion reported having infrequent contact with internet friends compared to individuals with no AL.

The range in RR’s was 0.97–2.23 among individuals with some AL and 0.95–3.84 among individuals with profound AL. Most of the RR’s were in the order of 2–3. Examples are infrequent contact with family and feeling alone with RR’s (95% CI) of 1.27 (1.06–1.53) and 2.23 (1.91–2.60) for individuals with some AL, and 1.77 (1.46–2.15) and 3.84 (3.15–4.68) for individuals with profound AL.

There was a trend towards less contact with social relations with more AL.

## Discussion

This study illustrated that a large proportion of Danish adults with AL experienced a poorer health and well-being, had an unhealthier lifestyle and poorer social relations than adults without AL, and there seemed to be a trend towards higher risk of poor health with more pronounced AL.

The Danish Health and Morbidity Surveys have the strengths of National representativity with random selection of potential participants through National registers, the large number of participants and the large amount of data on participants’ health and well-being, health behaviour, social relations and use of the health care system. Furthermore, the surveys include information on over-the-counter medicine which is not available from the Registry of Medical Statistics. The specific strengths of this health profile are the large number of health indicators and participants (both participants with profound, some and no AL) and that the questionnaire was also sent to institutionalized individuals. Furthermore, central questions were international and validated standard measures (for example self-perceived health [33], Cohen’s Perceived Stress Scale [34], the Saltin-Grimsby Physical Activity Level Scale [35], diseases [36]), and for other indicators, standard cut-points were used (overweight and obesity, alcohol limits, physical activity).

One of the potential weaknesses of this study is that only 57% of the invited chose to participate and although this participation rate is relatively high compared to other and similar studies, there is still a risk of underestimating the prevalence of AL, if individuals with AL were less likely to answer the questionnaire. There is also a possibility of selection if some of the potential participants were not able to answer the questionnaire due to physical, cognitive, or psychological limitations. The weighting for non-participation has dealt with some of this underrepresentation, but it is not possible to control for absent groups. Last, since the question on AL referred to health problems or disease, the relative risks might have been smaller if the question referred to general AL. It is emphasized that the study design does not allow differentiation between AL caused by an unhealthy lifestyle (health behaviour), and unhealthy lifestyle caused by AL.

The results of the few studies on the health of people with disabilities are remarkably consistent; in particular, the results on overall self-rated health are very strong [16, 21]. This is very similar to our finding that individuals with profound AL are three times more likely to experience poor self-perceived health. In our study, only alcohol and fast food were inversely and not consistently associated with AL, respectively. A possible explanation for the result that only among individuals with some AL, a higher proportion of individuals had a weekly intake of fast food could be accessibility – that individuals with profound AL do not have the same access to fast food as individuals with some or no AL. Regarding alcohol, the explanation could also be accessibility, but it could also be possible interference with medication or mobility. This result is supported by both the US Behavioral Surveillance System Study and the Korean study by Ko et al. [19, 20] showing that AL or disability was associated with lower odds of binge drinking [19] and not associated with binge drinking, respectively [20].

We also found that individuals with AL have a less satisfying sexual life than individuals with no AL. This is consistent with results from the US Behavioral Surveillance System Study where Kinne et al. found a lack of romantic relationships among people with disabilities [13]. Kinne et al. also reported more fatigue, sleeping problems, periods of depression, problems seeing/making friends and feelings of being isolated among people with disabilities as compared to people with no disabilities [13]. These findings are very similar to our findings on the same indicators.

However, the most relevant comparisons are probably studies in neighbour countries. In Sweden and Norway, the health of people with disabilities has only been published in native language reports from 2008 and 2010, respectively [37, 38]. The studies used data very similar to the data in our study, and although the studies did not include as many health indicators as the present study, the results were very similar and showed that individuals with functional disability had markedly poorer health status, health behaviour, and social contact than the general population. The relative risks of poor health were very similar to the relative risk found in our study, and furthermore, the prevalence of a high alcohol intake was lower among individuals with disability as compared to the general population [37, 38]. For example, in the Norwegian report, the risk of feeling alone was 4.4 and 3.4 for women and men, respectively, and the risk of obesity was 1.6 in both women and men.

It should be noted that the studies used different measures of disability. However, since disability is an umbrella term, covering bodily impairments (functional limitations), activity limitations, and participation restrictions, these measures, and combinations of them, all measure aspects of disability.

## Conclusions

Our study shows that even in a Nordic welfare system like the Danish, the relative health of people with disabilities is very similar to the relative health of people with disabilities in the US. Striking is also the fact that preventable aspects of health and health behaviour are worse among individuals with AL compared to individuals with no AL. According to the WHO ‘Equity in health implies that ideally everyone should have a fair opportunity to attain their full health potential, and more pragmatically, that none should be disadvantaged from achieving this potential, if it can be avoided’ [1]. A range of factors influence health status and the barriers persons with disabilities meet in their environment, including individual factors, living conditions, general socioeconomic, cultural and environmental conditions, and access to health care services [1]. Equality in health therefore inevitably involves the concept of accessibility in a wide perspective and can only be obtained by taking multiple approaches including social equality (inclusion), physical accessibility (physical environment and transport), an outreaching, pro-active and inclusive health care system, and education and awareness/acknowledgement among healthcare personnel. In relation to policy and legislation this specifically implies that discrimination is stopped and that people with activity limitation are included and prioritized in public health strategies. Furthermore, efforts should be done to secure the availability and flexibility of health care services and primary prevention programs; the environment (societies and communities) should be designed and arranged to facilitate the inclusion of people with activity limitation; and any intervention or service should be available close to the homes of people with activity limitation. Last, it should be ensured that people with activity limitation can afford the same standard of health care and health behaviour as other people.

## Additional file

Additional file 1: Table S1. Operationalization of health indicators. (DOC 46 kb)

## Abbreviations

AL: Activity limitation
BMI: Body mass index
CI: Confidence interval
RR: Relative risk
