# Supplementary material for: Health profile for Danish adults with activity limitation: a cross-sectional study
Source: BMC Public Health. 2017 Jul 24;18:46. doi: 10.1186/s12889-017-4532-0 (PMC5525283; doi:10.1186/s12889-017-4532-0)
Supplement: Additional file 1: Table S1. — Operationalization of health indicators. (DOC 46 kb) [file 12889_2017_4532_MOESM1_ESM.doc]

Additional file 1: Table S1. Operationalization of health indicators

| **Health indicator** | **Operationalization** |
| --- | --- |
| **Health and well-being** |  |
| 1. Poor self-perceived health | ”Less good” and ”Poor” versus ”Excellent”, ”Pretty good” and ”Good” |
| 1. Poor dental status | ”No teeth left”, ”1-9 teeth left” and ”10-19 teeth left” versus ”20 or more teeth left” |
| 1. Pain or discomfort in musculoskeletal system | ”Yes, very affected” and ”Yes, little affected” within the last 14 days versus ”No, not affected” |
| 1. Headache | Ditto |
| 1. Fatigue | Ditto |
| 1. Dyssomnia | Ditto |
| 1. Psychological symptoms (depressive, anxious, nervous or unrestful symptoms) | Ditto |
| 1. Stress | Cohen’s perceived stress scale ≥ 18 |
| 1. Hypnotics/sedatives (prescription-only) | Have taken the medicine within the last 14 days versus not |
| 1. Hypnotics/sedatives (over-the-counter) | Ditto |
| 1. Analgesics (prescription-only) | Ditto |
| 1. Analgesics (over-the-counter) | Ditto |
| 1. Laxatives (prescription-only) | Ditto |
| 1. Laxatives (over-the-counter) | Ditto |
| 1. No sexual contact | ”No” versus ”yes” to Sexual contact within the past year |
| 1. Unsatisfied with sexual life | “Unsatisfied” and “Highly unsatisfied” versus “Neither satisfied nor unsatisfied”, “Satisfied”, “Highly satisfied” and “Don’t know” |
| **Health behaviour** |  |
| 1. No daily intake of vegetables | Intake of vegetables less than once a day versus more often |
| 1. Weekly intake of fast food | Intake of fast food at least once a week versus less often |
| 1. Daily smoking | ”Yes, I smoke every day” versus ”Yes, at least once a week”, ”Yes, less than once a week”, ”No, I have stopped” and ”No, I have never smoked” |
| 1. Heavy smoking | Smoke at least 15 cigarettes a day versus fewer |
| 1. Alcohol intake above 7/14 units/week | Intake above 7 units/week among women and 14 units/week among men versus below these limits |
| 1. Alcohol intake above 14/21 units/week | Intake above 14 units/week among women and 21 units/week among men versus below these limits |
| 1. Sedentary leisure time activity | ”Reading, watching TV or having other sedentary behaviour” versus ”Walking, cycling or doing other light activity at least four hours per week”, ”Doing recreational sports or carrying out heavy gardening or similar at least 4 times per week” and “Training hard or doing competitive sports frequently and several times per week” |
| 1. Overweight | Overweight: BMI≥25 |
| 1. Obesity | Obesity: BMI≥30 |
| **Social relations** |  |
| 1. Infrequent contact with family | ”Less than once a month” and ”Never” versus ”1 or 2 times a month”, ”1 or 2 times a week” and ”Daily or almost daily” |
| 1. Infrequent contact with friends (incl. neighbours, colleagues, fellow students) | Ditto |
| 1. Infrequent contact with internet friends | Ditto |
| 1. Feeling alone | ”Yes, often” versus ”Yes, once in a while”, ”Yes, but seldom” and ”No” |
| 1. No one to talk to | ”No, never or almost never” versus ”Yes, often”, ”Yes, most of the time” and ”Yes, some times” |
| 1. No practical help | ”No” versus ”Yes, absolutely” and ”Yes, maybe” |
